# Skeletal muscle metastasis from a gastrointestinal stromal tumor: A case report

**DOI:** 10.1097/MD.0000000000027011

**Published:** 2021-08-27

**Authors:** Guangsheng Zhu, Wenjia Sun, Yujun Liu, Huabin Wang, Shengwei Ye

**Affiliations:** a Department of Gastrointestinal Surgery, Hubei Cancer Hospital, Tongji Medical College, University of Science and Technology Huazhong, Wuhan, China; b Department of Pathology, Hubei Cancer Hospital, Tongji Medical College, University of Science and Technology Huazhong, Wuhan, China; c Department of Bone and Soft Tissue Surgery, Hubei Cancer Hospital, Tongji Medical College, University of Science and Technology Huazhong, Wuhan, China.

**Keywords:** gastrointestinal stromal tumors, skeletal muscle metastasis, whole exome sequencing

## Abstract

**Rationale:**

Gastrointestinal stromal tumors (GISTs) are the most common mesenchymal tumors of the gastrointestinal tract. Common sites for metastasis are the liver and peritoneum, whereas skeletal muscle metastases are rare.

**Patient concerns:**

A 59-year-old man with skeletal muscle metastasis was diagnosed during a period of adjuvant imatinib therapy following the recurrence of GIST of the small intestine.

**Diagnosis:**

The patient was diagnosed with skeletal muscle metastasis of GIST based on immunohistochemistry and molecular pathology analysis results.

**Intervention:**

Extensive resection of the left thigh tumor was performed. The patient underwent whole-exome sequencing of tissue examination. The results suggest that resistance to imatinib may have been developed, and the patient was therefore administered sunitinib instead.

**Outcomes:**

Complete remission was observed following sunitinib therapy.

**Lessons:**

In cases of skeletal muscle metastasis diagnosed during a period of adjuvant imatinib therapy following the recurrence of a GIST of the small intestine, whole exome sequencing may be used to discover more gene variations.

## 1 Introduction

Gastrointestinal stromal tumors (GISTs) are the most common mesenchymal tumors of the gastrointestinal tract. The biological behavior of GISTs can range from benign to malignant. Most GISTs differentiate towards the interstitial cells of Cajal and have an activating mutation in the gene encoding KIT or platelet-derived growth factor receptor alpha (PDGFRA) tyrosine kinase.^[1]^ Although GISTs can develop anywhere along the gastrointestinal tract, 60% to 70% occur within the stomach and 20% to 30% occur in the small intestine.^[2–4]^ GISTs remain an unpredictable entity: almost 50% of patients develop recurrence or metastases within 2 years of presentation,^[5,6]^ and the most common metastatic sites are the liver and peritoneum.^[7]^ Metastasis to other sites, such as lymph nodes and skeletal muscles, is extremely rare.^[8–13]^ We present the case of a 59-year-old man with skeletal muscle metastasis diagnosed during a period of adjuvant imatinib therapy following the recurrence of a GIST of the small intestine. The patient provided informed consent for the publication of this case report.

## 2 Case report

A 59-year-old man was admitted to the hospital following the discovery of a tumor in the left thigh 1 month previously. The patient underwent a partial jejunal resection of a jejunal tumor in the outer hospital on June 13, 2017. Postoperative pathology revealed a jejunal GIST measuring 7 × 6 × 4 cm. Recurrence risk classification was high and results of the immunohistochemical analysis were CD117 positive, CD34 positive, DOG-1 positive, SMA negative, S-100 negative, SDHB positive, and Ki67 LI 5%. Molecular pathology analysis revealed that the C-KIT gene exon 13 had a mutation in p.K642E (c.1924A>G), while no PDGFRA gene mutation was detected. The patient was started on imatinib (oral 400 mg/day) treatment after surgery. The treatment was well tolerated, with no grade 3 adverse events. On August 28, 2018, a follow-up abdominal computer tomography in the outside hospital showed an ileocecal mass, which was considered to be a tumor recurrence. He was referred to our hospital for treatment, and on September 12, 2018, ileocecal tumor resection was performed. Postoperative pathology confirmed the tumor to be a necrotic and bleeding ileocecal GIST measuring 5.5 × 5 × 5 cm with a mitotic rate of >5 mitoses per 50 high-power fields (HPFs). The results of the immunohistochemical analysis were CD117 positive, CD34 negative, DOG-1 positive, SMA negative, S-100 negative, and Ki67 LI 20%. Molecular pathology analysis revealed that the C-KIT gene exon 13 had a mutation in p.K642E (c.1924A>G), while no PDGFRA gene mutation was detected. Postoperatively, the patient was orally administered imatinib mesylate 400 mg/day.

**Figure 1:**
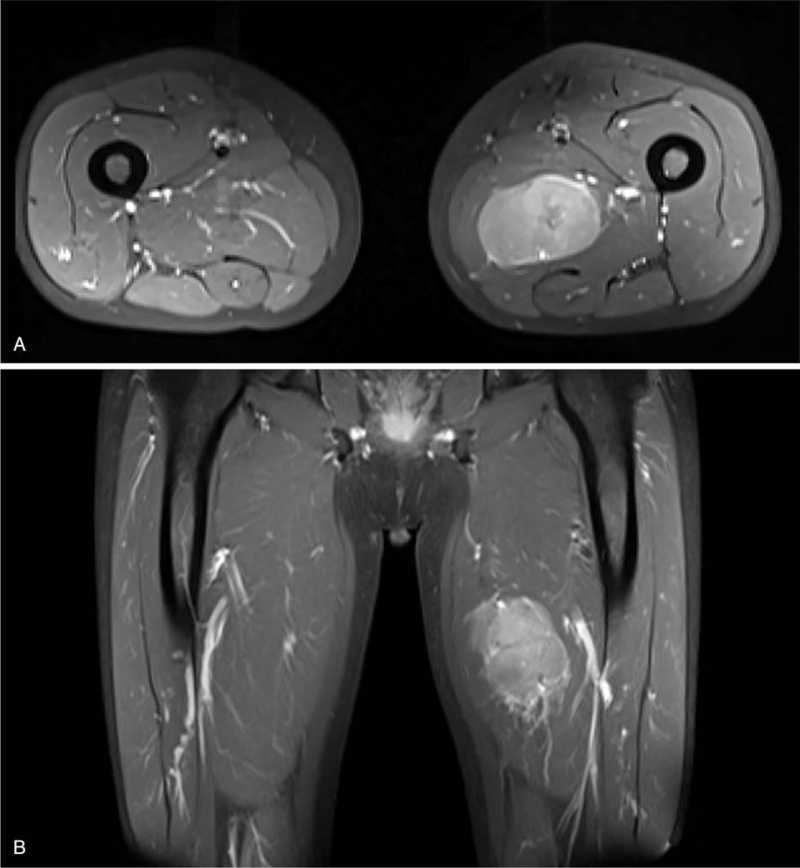
Axial (A) and coronal (B) T1 views of an MRI of the lesion in the context of the left medial thigh muscle. MRI = magnetic resonance imaging.

**Figure 2:**
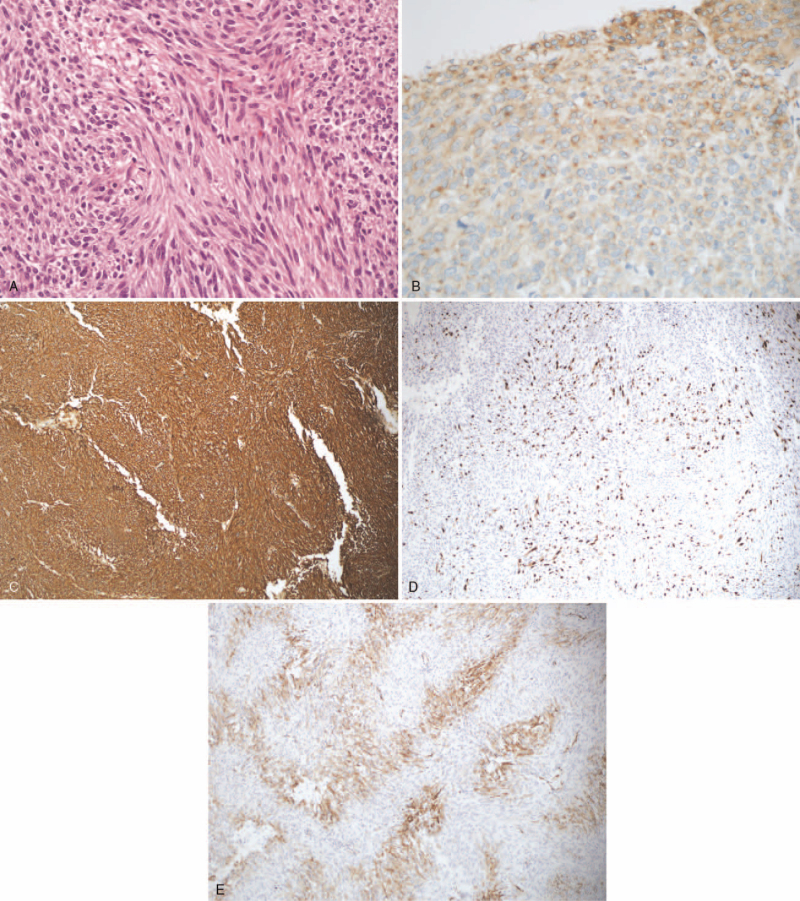
HE and IHC images of this tumor, (A) epithelioid cell type (HE, ×400), (B) IHC staining of CD117 positive (IHC, ×400), (C) IHC staining of DOG-1 positive (IHC, ×100), (D) IHC staining of SMA positive (IHC, ×100), (E) IHC staining of Ki-67 proliferation index (IHC, ×100). HE = hematoxylin-eosin staining, IHC = immunohistochemistry.

The patient was admitted to the hospital with a mass on the left thigh that had been gradually increasing in size for 1 month. Lower limb magnetic resonance imaging showed a left medial thigh muscle mass measuring 7.4 × 5.7 × 3.7 cm and with a clear boundary (Fig. 1). Considering the history of a previous mesenchymal tissue tumor, metastasis could not be excluded. Extensive resection of the left thigh tumor was performed on September 3, 2020. Postoperative pathology revealed a left thigh sarcoma with focal necrosis. Histopathological examination revealed a hypercellular neoplasm. The neoplastic cells were arranged in either a fascicular or whirling pattern and exhibited spindle and epithelioid morphology (Fig. 2). Immunohistochemistry analysis showed that CD117 foci were positive, DOG-1 positive, CD34 negative, SMA focus positive, desmin negative, S-100 negative, PCK negative, and Ki67 LI 45%. Based on the combination of morphology, immunohistochemical results, and medical history, this was found to be consistent with GIST metastasis. No tumor cells were observed around the margins. Molecular pathology analysis revealed that the C-KIT exon 13 had a mutation in p.K642E (c.1924A>G). This patient underwent whole exome sequencing for tissue examination, and the results are listed in Table 1. There are 9 tumor core gene variations, including the KIT gene missense mutation in p.K642E (c.1924A>G) and p.T670I (c.2009C>T), KIT gene copy number gains, and PDGFRA gene copy number gains. These results suggest that resistance to imatinib may have been developed, and the patient was administered sunitinib orally 37.5 mg/day after the third surgery. Meanwhile, the treatment was well tolerated, with no grade 3 adverse events. Until May 3, 2021, the patient was still under treatment, and no tumor recurrence or metastasis was found.

**Table 1:** The results of whole exome sequencing.

Gene	Transcript	Result	Mutational abundance or copy number	Type of mutation
KIT	NM_000222.2	p.K642E (c.1924A>G)	79.76%	Missense mutation
NF2	NM_000268.3	p.K69Rfs∗54 (c.204delC)	55.64%	Frameshift mutation
KIT	NM_000222.2	p.T670I (c.2009C>T)	15.11%	Missense mutation
KIT	NM_000222.2	Copy number gains	7.05	Copy number gains
PDGFRA	NM_006206.4	Copy number gains	5.7	Copy number gains
KDR	NM_002253.2	Copy number gains	5.33	Copy number gains
CDKN2A	NM_000077.4	Copy number losses	0.35	Copy number losses
MAX	NM_002382.4	p.V99F (c.295G>T)	75.33%	Missense mutation
RPS6KA4	NM_003942.2	p.S421R (c.1263C>G)	42.46%	Missense mutation

## 3 Discussion

A significant feature of GISTs is that tumor recurrence and metastases are prone to occur following surgery. The anatomic site, tumor size, and mitotic activity are among the most relevant factors proposed to predict GIST behavior.^[14,15]^ GIST recurrences usually occur within the first 5 years following primary surgery, and recurrence-free survival at 5, 10, and 15 years is estimated to be 70%, 63%, and 60%, respectively.^[16]^ Common sites for metastasis include the liver, peritoneum, and omentum.^[17]^ Metastases to lymph nodes and extra-abdominal structures are uncommon, occurring in less than 10% of cases.^[18]^ Skeletal muscle metastasis from a primary GIST is exceptionally rare, and to our knowledge, only 6 cases have been reported in the literature.^[8–13]^ Pasku et al^[8]^ reported a case of bilateral gluteal and lung metastases in a 56-year-old woman with a GIST of the pelvis, which was initially misdiagnosed as a uterine leiomyosarcoma. She was successfully treated with tumorectomy and adjuvant imatinib for 1 year. Bashir et al^[9]^ presented the case of a 56-year-old man with upper back muscle, adrenal gland, and cardiac metastases from a GIST of the small intestine. The tumor was excised, and the patient received tyrosine kinase inhibitor (TKI) therapy, although the final outcome was not reported. Suzuki et al^[10]^ reported the case of a 54-year-old man who presented with an enlarging mass of the left buttock initially misdiagnosed as a leiomyosarcoma. Following surgical excision and immunohistochemical analysis of the tumor, the patient was ultimately diagnosed with GIST of the small intestine with skeletal muscle metastasis. Despite treatment, the patient died of overt gastrointestinal bleeding 6 months after the diagnosis. Cichowitz et al^[11]^ reported the case of a 23-year-old woman with a 6 cm lesion in the adductor longus muscle presenting 5 years after the initial resection of an 11 cm small bowel GIST. She underwent resection of the lesion, and the analysis confirmed the diagnosis of muscle metastasis from the primary GIST. Jin et al^[12]^ reported the case of an 80-year-old woman who presented with a soft tissue mass in the setting of a gastric GIST with an omental invasion that had been diagnosed 3 years earlier. An incisional biopsy confirmed the presence of a solitary metastatic muscle mass. Due to the patient's unstable state of health, the lesion was not excised, and she received TKI therapy. Finally, Savvidou et al^[13]^ reported the case of a 78-year-old man with medial right upper thigh metastases from an anal GIST. The patient subsequently underwent wide excision of the lesion, which confirmed the diagnosis of skeletal muscle metastases secondary to GIST.

Of the 6 known cases of muscle metastases, 3 were from a GIST of the small intestine. Although most GISTs occur in the stomach, obvious malignant behavior is rarely observed in gastric tumors.^[14]^ In contrast, tumors of small bowel origin tend to be more aggressive than tumors from other gastrointestinal sites, and therefore have a worse prognosis.^[14,19]^ According to the standards of the National Institutes of Health, tumor size and the number of mitoses per HPF are predictors of GIST recurrence.^[20]^ Moreover, >5 mitoses per 50 HPF and tumor size >5 cm are both associated with an increased risk of recurrence.^[3]^ Therefore, the patient described in this report had an extremely aggressive GIST with a high likelihood of recurrence: mitosis count >5 per 50 HPF, and tumor size >5 cm.

The 3 molecular pathological examinations of the tumor specimens of this patient showed that the C-KIT gene exon 13 had a mutation of the type c.1924A>Gp (Lys642Glu). The C-KIT exon 13 mutation is a common secondary mutation in GISTs and accounts for a relatively low proportion. It is reported in the literature 0.6% to 4.4%, and it is also one of the mechanisms of secondary drug resistance.^[21–23]^ Secondary resistance refers to the remission or stability of initial treatment with TKIs, and the progression of tumors as the treatment time increases. The mechanism of secondary resistance is generally the development of a secondary gene mutation in the C-KIT gene or PDGFRA. This usually occurs in the ATP-binding site (exons 13 and 14) and the receptor activation loop (exons 17 and 18).^[24]^ A meta-analysis showed that secondary KIT mutations associated with imatinib secondary resistance occurred most frequently in exon 17 (54.5%), followed by exons 13 (38.3%) and 14 (13.4%).^[25]^ Our patient had a C-KIT exon 13 mutation before receiving TKI, which is considered to be a primary mutation.

Whole exome sequencing of tumor specimens in this patient showed a KIT gene missense mutation in p.K642E (c.1924A>G) and p.T670I (c.2009C>T), KIT gene copy number gains, and PDGFRA gene copy number gains. A clinical trial recruited 97 patients with metastatic GIST who were resistant or intolerant to imatinib for treatment with sunitinib. Among them, 78 patients were tested for KIT/PDGFRA mutation status. The results showed that one of the patients had the C-KIT 13 exon K642E primary mutation and the exon 17 D816H secondary mutation and had received sunitinib for stable disease for more than 6 months.^[26]^ The literature also confirms that sunitinib is effective for tumors with C-KIT exon 13 or 14 mutations.^[27,28]^ The KIT protein and RNA levels in GISTs were highly variable, but they were closely related (*r* = 0.82, *P* < 1.10(–5)), and the KIT protein and RNA levels mutated in GISTs were higher (*P* = .07, *P* = .03, respectively). In addition, the overexpression of KIT in GISTs is rarely associated with gene amplification.^[29,30]^ Studies have reported that overexpression of the wild-type PDGFRA gene can activate AKT and ERK to achieve an optimal level of tumorigenicity. Another study reported that an increase in the PDGFRA gene copy number is related to a poorer overall survival rate (*P* = .027).^[31,32]^

Incidentally, additional gene mutations were detected in tissue examination including neurofibromatosis 2 gene frameshift mutation, KDR gene copy number gains, CDKN2A gene copy number gains, MAX, and RPS6KA4 genes missense mutation. The neurofibromatosis 2 tumor suppressor protein merlin or schwannomin inhibits cell proliferation by regulating its growth activity in binding chaperones. Studies have shown that in thyroid cancer, neurofibromatosis 2/merlin inactivation enhances RAS signal transduction by promoting YAP/TEAD-driven oncogenic and wild-type transcription, leading to enhanced MAPK output and greater sensitivity to MEK inhibitors.^[33,34]^ However, KDRS, CDKN2A, MAX, and RPS6KA4 are rarely reported to be related to the occurrence and treatment of GISTs, which requires further study.

In conclusion, while there are few reports of skeletal muscle metastases arising from GISTs with a significant improvement in the progression-free survival rate and overall survival rate of GISTs, the possibility of metastases at unusual sites is likely to increase. In addition, the side effects of TKIs are very common; therefore, skeletal muscle metastases should be actively removed by surgery.

## Acknowledgments

We are particularly grateful to all the people who have provided us with help in our article.

## Author contributions

Guangsheng Zhu wrote the first draft of this manuscript. Shengwei Ye substantially revised the manuscript and approved its final version. Wenjia Sun, Yujun Liu, and Huabin Wang participated in patient care.

**Methodology:** Wenjia Sun.

**Resources:** Yujun Liu, Huabin Wang, Shengwei Ye.

**Writing – original draft:** Guangsheng Zhu.

**Writing – review & editing:** Shengwei Ye.
